# Past attachment experiences, the potential link of mentalization and the transmission of behavior to the child by mothers with mental health problems: cross-sectional analysis of a clinical sample

**DOI:** 10.1007/s00787-023-02291-9

**Published:** 2023-09-05

**Authors:** Janna Mattheß, Gabriele Koch, Thomas Keil, Stephanie Roll, Anne Berghöfer, Christiane Ludwig-Körner, Franziska Schlensog-Schuster, Mona Katharina Sprengeler, Kai von Klitzing, Lars Kuchinke

**Affiliations:** 1 International Psychoanalytic University, Stromstr. 3B, 10555 Berlin, Germany; 2 Department of Child and Adolescent Psychiatry, Psychotherapy and Psychosomatics, University of Leipzig, Leipzig, Germany; 3 Institute of Social Medicine, Epidemiology and Health Economics, Charité-Universitätsmedizin Berlin, Berlin, Germany; 4 Institute of Clinical Epidemiology and Biometry, University of Wuerzburg, Würzburg, Germany; 5 State Institute of Health I, Bavarian Health and Food Safety Authority, Erlangen, Germany; 6 University Hospital of Child and Adolescent Psychiatry and Psychotherapy, University of Bern, Bern, Switzerland

**Keywords:** Reflective functioning, Attachment, Mother–child interaction, Paternal attachment experiences, Transgenerational transmission

## Abstract

Maternal capacity to mentalize (= reflective functioning, RF), secure attachment and emotionally available parenting has an impact on the child’s development. The transmission of mothers’ past attachment experiences gained with both her caregivers in her own childhood and the impact on current mother–child interaction is part of the ‘transmission gap.’ This study explores the transgenerational transmission mechanisms and the potential moderating effect of RF in a clinical sample of 113 mother–child dyads suffering from mental health problems. In a cross-sectional study, the associations between maternal attachment experiences, RF (coded based on Adult Attachment Interviews) and current mother–child interaction (Emotional Availability Scales) were examined with univariate correlation, moderator analyses, and structural equation models. We found relationships between attachment experiences and mother–child interaction, but RF had no moderating effect. Past loving experiences and perceived neglection, particularly with the own father in childhood, were predictors for the present mother–child interaction. There seems to be an intergenerational transmission of attachment experiences to the ongoing generation. Particularly past adverse childhood experiences with the own father seem to explain currently disruptive interactions with the child.

*Trial registration:* DRKS00017008 and DRKS00016353.

**Supplementary Information:**

The online version contains supplementary material available at 10.1007/s00787-023-02291-9.

## Introduction

Over the past decades, an impact of a secure parental attachment representation, i.e., the parental capacity to mentalize and the parent–child interaction on the development of the child was reported [1, 2]. For example, the impact of mothers suffering from mental health problems and their experiences had in their own childhood as well as their effects on current behaviors toward the own infant and the child’s development is part of the so-called ‘transmission gap’—the gap between what is known and not known to explain the mechanisms that support a secure attachment development [2]. Until now, research mainly focused on the mother–child dyad, excluding the father as third person [3].

A child has an inborn need for proximity and care to survive which, according to attachment theory, can be satisfied by a responsive and sensitive caregiver [4]. Parents who had sufficiently good attachment experiences with their own parents are more likely to understand and help the child’s needs and to serve as a secure base for the child's attachment development. This ability is especially helpful in stressful situations and leads to less disrupted dyadic interactions and to a child who is more sensitive in his/her responses in return [5]. Negative childhood experiences are considered risk factors for later psychopathology, whose prevalence in turn can be predicted by the frequency, severity and cumulation of these negative childhood experiences. The development of early relationships and the child’s mental development depends on at least three individuals (most typically mother, father, and child) [6]. Accordingly, it seems important to differentiate between attachment experiences with each caregiver, respectively. The Adult Attachment Interview (AAI) [7] provides an independent and reliable external judgment of attachment classifications, and the subscales differentiate between childhood attachment experiences with the mother and the father. The AAI can be used to identify whether a caregiver was loving, rejecting, neglecting, involving or put pressure on to achieve [7]. It is assumed that these past experiences are subsequently transmitted to the ongoing relationship with the child.

Several studies have shown that parents with insecure or unresolved attachment representations show less understanding of their own and other’s feelings and intentions [8], leading to insensitive up to hostile behavior toward the child. Insensitive maternal behavior toward the child is known to correlate with childrens’ attachment security [1]. A lack of parental responsiveness in the dyadic interaction is considered to be a potential risk for infant abuse and psychological distress. Especially parents who have experienced this kind of behavior in their own childhood are known to transmit these malignant patterns to the child [9, 10]. Children who suffered from a rejecting parent have a higher likelihood of developing mental health problems like social or adjustment impairments, emotional problems or becoming a rejecting parent as well [11–14]. Self-reported hostile experiences with both caregivers, mother and father predict hostile behavior in the next generation [15].

Attachment and sensitivity have long been discussed as the only mechanisms of intergenerational transmission of attachment. Recent research highlights the concept of reflective functioning (RF) to also play a vital role in the transmission [16]. RF is the operationalization of mentalization [5], i.e., the capacity to envision mental states in oneself or another. RF can be assessed by the RF rating scale in the context of the AAI. The RF rating scale objectively codes the awareness of mental states characteristics, the effort to tease out the underlying behavior of mental states and/or the recognition of their developmental aspects [17]. As such, a close relationship between the AAI experiences subscales and RF could be predicted, but concrete evaluations, for example, in relation to each caregiver are lacking. It is hypothesized that RF can be a protective factor against parents’ insensitive behavior: Parental RF has been identified as one of the key factors in a positive parent–child relationship. It predicts sensitive parenting and attachment security and results in a decrease of child behavioral problems [16, 18]. Moreover, mothers with higher RF show less hostility and intrusiveness in the interaction with their children [16]. On the other hand, low parental RF is associated with risk factors like mental health problems, chronic stress or unresolved trauma or loss. In turn, parental mental health problems are known risk factors for infant psychopathology [19]. Especially in stressful situations with the child, the ability to provide consistent and sensitive caregiving can be impaired which increases the risk for transmission [13]. Parents with low RF are significantly more likely to demonstrate less sensitive caregiving behavior by showing a lack of awareness of the infant or an inaccuracy in interpreting the infant’s internal states [1].

In sum, links between sensitive caregiving, attachment and RF already exist [10, 18]. However, evaluations of the relationship between past attachment experiences, for example, with a loving, rejecting or neglecting caregiver and RF are lacking. It is still uncertain how these patterns influence the transgenerational transmission and whether there exist further mechanisms explaining the transmission gap. The observed relationship between sensitive caregiving, mentalization and child attachment explains only up to 12% of the variance in infant attachment security [1]. Thus, it seems likely that further transmission mechanisms exist. Based on cross-sectional data from a clinical sample with maternal and/or infant mental health problems, this study aims to evaluate potential mechanisms of the transmission gap by examining the potential link between mother’s attachment experiences with her caregivers in the past and her current behavior toward the child. If such a link is confirmed, the potential moderating effects of maternal RF on this relationship will be evaluated.

## Method

### Data set

The data are taken from two randomized controlled trials (RCTs) of the German multicenter research project SKKIPPI ['SKKIPPI’, [20–22]. The RCTs were designed to evaluate the efficacy of Parent–Infant Psychotherapy (PIP) to improve maternal mentalization and mother–child interaction in early childhood. The final sample size in both RCTs was *N* = 260. The aims of the two RCTs were to evaluate the efficacy of dyadic PIP in inpatient psychiatric units and outpatient settings. To avoid interference with ongoing trials, only a subsample of mother–child dyads with a full dataset of baseline assessments have been included in the present analyses. Data of this subsample analysis have been recorded between January 2019 and September 2021 (data collection in the SKKIPPI RCTs ended in December 2022).

### Participants

A subsample of *n* = 113 mother–child dyads were part of the present analyses. Mother–child dyads were included when complete baseline data for EAS and AAI (attachment experiences and AAI-RF) outcomes were available. Dyads were included in data analysis, independent of later RCT treatment allocation or psychopathological symptoms. Mothers in the RCTs either had a current ICD-10 diagnosis of a mental disorder in the postpartum period or children received an ICD-10 diagnosis of regulatory disorder. Mothers (*M* = 32.7 years) were recruited in 5 German study centers together with their children (44.2% girls; see Table 1). Due to the inclusion criteria of the SKKIPPI RCTs, child age ranged from 0 to 36 months. 61.1% of mothers were found to have a current mental health problem according to the M.I.N.I. diagnostic interview [23] (see Table 1). Mother–child dyads with a maternal ICD-10 diagnosis of schizophrenia, substance abuse, recent suicidal ideation, or infant’s symptoms of alcohol embryopathy or severe life-limiting diseases were excluded from study participation.

**Table 1:** Sociodemographic characteristics of participating mothers

	*n*	Mean	SD	Range	%
Mother’s age (years)	113	32.7	4.78	17–44	
Infant ‘s age (months)	113	12.0	10.9	0–36	
Sex of infant	113				
Male	63				55.8
Female	50				44.2
Born in Germany	83				86.5
Marital status	92				
Single	8				8.7
Living with a partner	84				91.3
Educational level	98				
Low	7				7.1
Medium	24				24.5
High	67				68.4
Number of children					
1	40				53.3
2	27				36.0
3	7				9.3
> 4	1				1.3
Mental health problems (M.I.N.I.)*	69				61.1
Depression	40				35.4
Manic or hypomanic episode	4				3.6
Panic disorder	30				26.5
Social phobia	15				13.3
OCD	12				10.6
PTSD	7				6.2
Eating disorder	2				1.8
GAD	34				30.1
ASPD	3				2.7
Attachment classification					
Secure (F)	46				40.7
Insecure-dismissive (Ds)	23				20.4
Insecure-entangled (E)	14				12.4
Disorganized (U/d)	23				20.4
Cannot classify (CC)	7				6.2

*n*  frequency, *SD*  standard deviation, *M.I.N.I. standardized diagnostic interview, multiple diagnoses of mental health problems for each participant were possible, *OCD*  obsessive compulsive disorder, *PTSD*  post-traumatic stress disorder, *GAD*  generalized anxiety disorder, *ASPD*  antisocial personality disorder

### Study procedure

After enrollment, informed consent to participate was given. At baseline, mothers were videotaped during a 15–20 min play interaction with the child to subsequently code maternal behavior toward the child by the Emotional Availability Scale (EAS) [24]. All mothers were also interviewed with the AAI to assess the maternal attachment classification, AAI-RF and attachment experiences in their own childhood. Only these direct assessments of EAS, past attachment experiences and AAI-RF are used in the present study. In the event of an interview or video that was difficult to code, the coders had the possibility of discussing the ratings with one another, but at the time of writing no inter-rater reliability (IRR) could be provided. Further self-reported outcomes that are not of interest for the present analyses have been assessed elsewhere [20, 21].

### Measures

A sociodemographic questionnaire and a diagnostic interview (M.I.N.I.) [23] were administered at baseline to gather information including maternal and child’s age, child’s gender, country of origin, and marital status as well as maternal mental health problems (Table 1).

#### Emotional Availability Scale

The EAS is a widely used assessment to measure the quality of mother–child interaction with excellent psychometric properties [24]. Recorded dyadic play interactions are coded by independent and reliable coders. EAS is measured on 6 scales each ranging from 1 to 7: Maternal Sensitivity, Structuring, Non-intrusiveness and Non-hostility, as well as child’s Responsiveness to parent and Involvement with parent. Maternal Sensitivity refers to a warm and responsive mother, while Structuring describes a mother who is supportive and guiding in the interaction with the child. Non-intrusiveness refers to a mother who follows the child’s lead, and Non-hostility describes a mother who is emotionally well-regulated and has a gentle tone. Child’s Responsiveness to parent indicates if a child is positive in affect and enjoying the interaction and involvement with parent refers to the degree that the child is balanced and engaged in play with the mother. The scale’s continuum refers to a parent who either does not show the behavior (= 1) or shows it to a high degree in play interaction with the child (= 7)[24]. Scores from 1–3.4 are commonly interpreted as indicating emotional availability in mother–child interactions ‘at risk,’ 3.5–4.5 as ‘some risk’ and 4.6–7 as ‘non-risk.’

#### Adult attachment interview

AAI is a semi-structured interview with excellent psychometric properties to evaluate the maternal ‘state of mind’ with respect to RF and the maternal attachment classification [25]. The AAI is conducted by trained interviewers, transcribed, and subsequently scored by reliable and independent coders (others than EAS coders in the present study). The AAI has been developed to reach an attachment classification score: Secure (F), Insecure-dismissive (Ds), Insecure-entangled (E) and Disorganized with respect to loss or trauma (U/d). A Cannot classify (CC) category is utilized when no attachment classification is predominant.

Relevant for the present study are attachment experiences with each important attachment figure (= caregiver) in their own childhood. These past experiences are captured by the AAI attachment experiences scales [25]. The five experience scales refer to Loving, Neglecting, Pressure to achieve, Involving and Rejecting behavior toward the child and are scored in relation to each important caregiver. The attachment experiences scales range from 1 to 9, portraying a parent who is either not showing this behavior (= 1) or showing it extremely (= 9). For instance, a low score on Loving scale means that a parent was not loving throughout the childhood, whereas a score of 9 refers to an extremely and overtly loving parent. The Neglecting scale describes a parent who although at home, was preoccupied with other things and Pressure to achieve was a parent who pushed the child to success with withdrawal of affection. Role reversal refers to a parent who demanded the involvement and/or attention of the child and may seek parenting from the child during childhood. The Rejection scale is considered when a child tried to attract attention but was pushed away by the parent. At the high end of the scale (= 9) the parent minimizes or ridicules the child’s expressions and needs and was portrayed as emotionally cruel or as actively disliking the child [see [25].

The AAI-RF scale was used to capture maternal mentalizing capacities represented by a global score ranging from − 1 to 9. A score of 9 is described as highly and exceptionally reflective and a − 1 as not at all reflective, overtly defensive, negative, or inappropriate. An AAI-RF score of 3 indicated a low or questionable RF and 5 is an ordinary RF and the most common rating in a community sample [17].

### Data analysis

All analyses were conducted using *jamovi* [26] on pseudonymized data. Descriptive and explorative statistics were computed to examine the study sample and the relationships among the variables of interest, followed by moderator analyses and a structural equation modeling (SEM) approach. As none of the AAI and EAS subscales followed a Gaussian distribution (according to Shapiro–Wilks tests, all *p*’s < 0.001), robust methods were applied. Wilcoxon rank tests and Spearman’s rank correlations will be reported, and moderator analyses will compute bootstrapped standard errors based on 1000 samples. Similarly, the reported SEMs have been computed with robust weighted least squares (WLSMV)[27] and robust standard errors as provided by *jamovi’s semlj* toolbox.

Pairwise correlations were computed to explore the relationship between EAS, AAI-RF, and attachment experiences with both caregivers. Subscales of EAS referring to maternal behavior toward the child (Sensitivity, Structuring, Non-intrusiveness and Non-hostility), the AAI attachment experience scales (Loving, Rejection, Neglection, Pressure to achieve and Involving), and the AAI-RF scale were included in this analysis. Subsequently, when a correlation at *p* ≤ 0.05 was revealed in the first step, past attachment experiences (as predictors) and the present interaction with the child (EAS subscales as criterion) were included in moderator analyses with AAI-RF (as moderator). Jamovi’s medmod toolbox computes moderator analyses based on a SEM model (developed from R’s lavaan toolbox). A moderation is indicated in these analyses when an interaction effect (predictor*moderator) on the criterion is revealed.

In a third exploratory step, SEMs were computed to further evaluate the relationships between past attachment experiences with the caregivers and current maternal behavior toward the child (EAS). Attachment experiences with each caregiver (past attachment experiences with mother = MAE and father = PAE) were modeled as latent exogenous variables that predict the present mother–child interaction (endogenous latent variable, EAS). To restrict the number of parameters in these SEMs, simpler models with only one observed indicator for MAE and PAE were computed first, while in a subsequent step, models with significant relationships between latent variables were combined into a final SEM. To evaluate model fit a *χ*^2^-test is computed as well as the fit indices CFI, RMSEA and SRMR (38). *χ*^2^ values close to zero and a nonsignificant *χ*^2^-test indicate good fit to data, as does a root mean square error of approximation, RMSEA, below 0.05 (below 0.08 is interpret as acceptable). A Comparative Fit Index, CFI, close to 1 with a cut-off of ≥ 0.90, and the value of the standardized root mean square residual (SRMR) 0.1 indicate a reasonable fit of the model [27].

## Results

### Descriptives

**Fig. 1:**
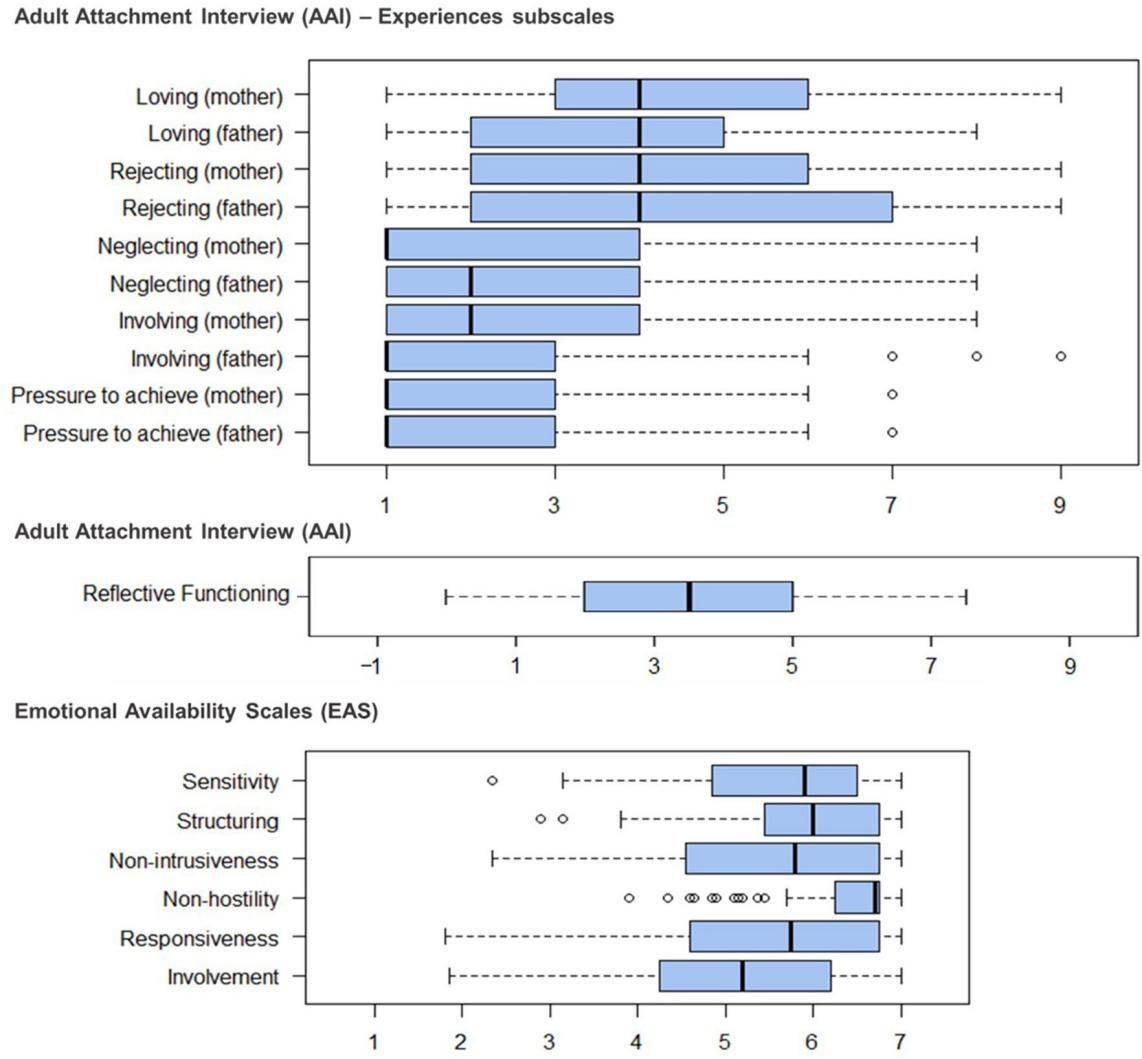
Boxplots indicating levels of Emotional Availability (EAS subscales), Reflective Functioning (AAI-RF) and past attachment experiences (AAI attachment experiences scales) in the sample

Participants had moderate to high scores across all EAS subscales (Fig. 1). In the AAI all *n* = 113 participants named their mother as primary caregiver (= C1) and *n* = 109 named their father (including stepfather, *n* = 4, and adoptive father, *n* = 1) as the second important caregiver (= C2). Other mentions for C2, that had to be excluded from respective analyses, included grandmother (*n* = 2), godfather and aunt (*n* = 1 each). Past attachment experiences scales showed low to average scores and AAI-RF ranged from questionable to average RF scores (Fig. 1 and Table 2).

**Table 2:** Descriptives of AAI attachment experiences scales for both caregivers (mother and father) and Wilcoxon rank tests comparing experiences gained with mother and father

	*N*	Mean	Median	SD	Wilcoxon W	*p* value
Loving mother	112	4.30	4.00	2.16	2316	0.002**
Loving father	107	3.82	4.00	1.86		
Rejecting mother	110	4.12	4.00	2.47	1299	0.166
Rejecting father	101	4.32	4.00	2.45		
Neglecting mother	109	2.46	1.00	1.96	471	0.018*
Neglecting father	95	2.92	2.00	2.15		
Involving mother	109	2.66	2.00	2.14	724	0.007**
Involving father	97	2.09	1.00	1.83		
Pressure to achieve mother	92	2.34	1.00	1.90	340	0.027*
Pressure to achieve father	93	2.01	1.00	1.65		

*N*  sample size, *SD*  standard deviation, *p < 0.05, **p < 0.01

### Past attachment experiences, maternal dyadic interaction and RF

Spearman’s rank correlations revealed associations between maternal EAS subscales of Sensitivity, Non-intrusiveness and Non-hostility with the attachment experiences scales Loving, Rejecting, Neglecting and Pressure to achieve for at least one of the caregivers. AAI-RF was also found to correlate with past Loving and correlated with Rejecting attachment experiences with mother and father, as well as Neglecting attachment experiences with mother and with EAS Sensitivity and Non-intrusiveness (Table 3).

**Table 3:** Pairwise correlation coefficients of EAS scales, attachment experiences scales and Reflective Functioning (AAI-RF)

AAI attachment experiences	EAS Scales				AAI-
	Sensitivity	Non-Hostility	Structuring	Non-Intrusiveness	RF
*Loving*					
Mother	0.290**	0.205*	0.153	0.204*	0.453***
Father	0.262**	0.278**	0.153	0.184 +	0.288**
*Rejecting*					
Mother	− 0.168 +	− 0.160 +	− 0.062	− 0.068	− 0.290**
Father	− 0.158	− 0.202*	− 0.047	− 0.081	− 0.224**
*Neglecting*					
Mother	− 0.211*	− 0.186 +	− 0.140	− 0.160 +	− 0.193*
Father	− 0.235*	− 0.267**	− 0.177 +	− 0.169	0.057
*Involving*					
Mother	0.045	− 0.095	0.081	0.018	0.080
Father	0.007	− 0.101	0.030	0.000	0.046
*Pressure to achieve*					
Mother	− 0.040	− 0.052	− 0.017	0.055	− 0.112
Father	0.101	0.060	0.018	0.214*	0.004
*AAI-RF*	0.193*	0.141	0.083	0.220*	

+ *p* < 0.10; *p < 0.05; **p < 0.01; ***p < 0.001

### Relationship between past attachment experiences and maternal dyadic interaction with RF as moderator

In the correlation analyses significant relationships between EAS subscales Sensitivity, Non-intrusiveness and Non-hostility and the attachment experiences scales Loving, Rejecting, Neglecting and Pressure to achieve in relation to mother and/or father were observed and therefore considered for further analyses (Table 3). Accordingly, ten moderator analyses were run to further examine these relationships with regard to RF. None of these analyses revealed a moderation effect (Supplement A).

### Predicted maternal dyadic interaction by past attachment experiences

**Fig. 2:**
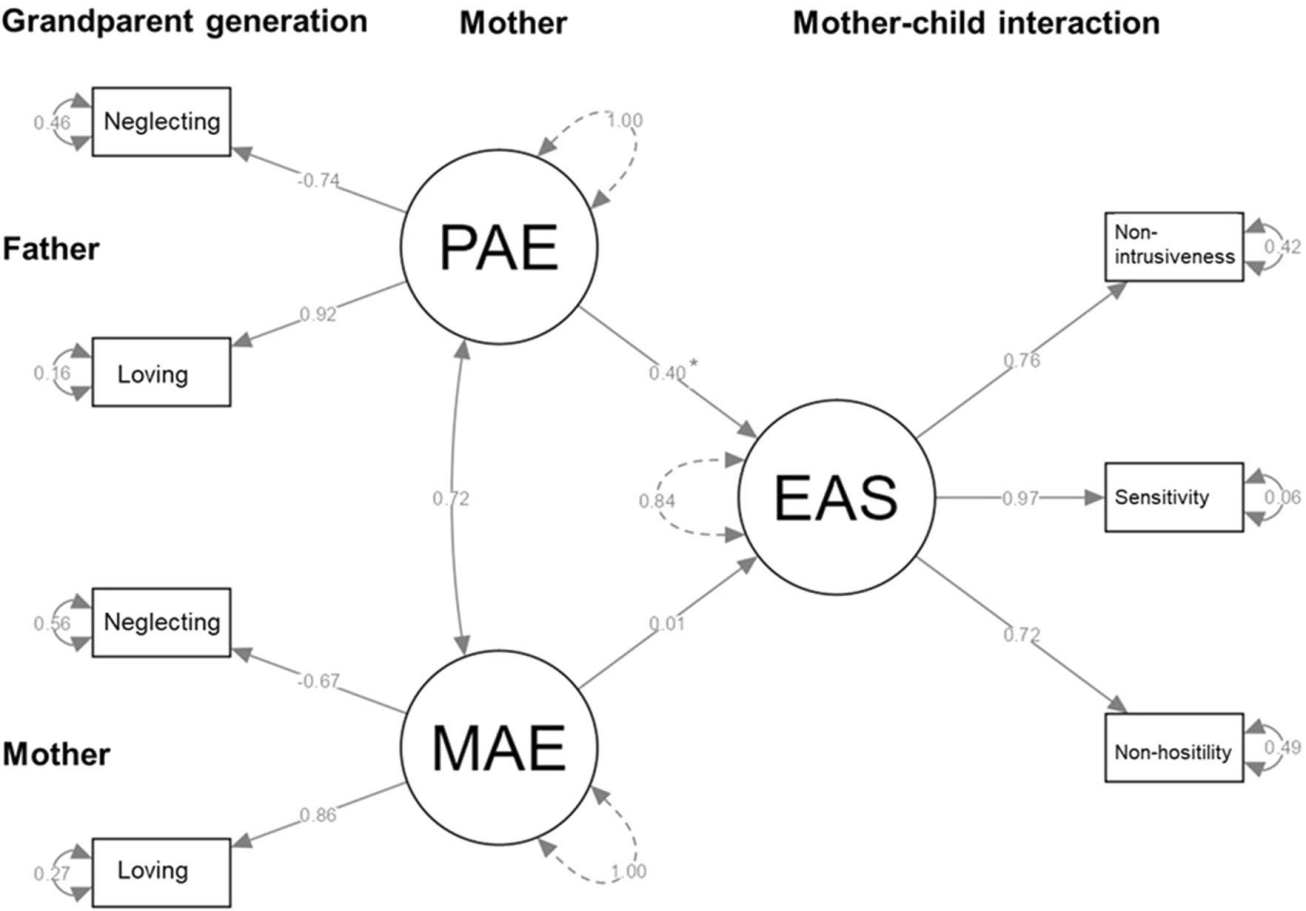
Structural equation model to predict (latent) maternal Emotional Availability (EAS) by attachment experiences with their own caregiver modeled as exogenous latent variables. Displayed are *β* estimates from SEM. PAE = AAI attachment experiences with father; MAE = AAI attachment experiences with mother. *p < 0.05

First, four SEMs were computed with one attachment experience scale (e.g., Loving experiences with mother (MAE) and with father (PAE), respectively, Supplement B) as single indicators of a latent exogenous variable and EAS subscales Sensitivity, Non-hostility and Non-intrusiveness as indicators of the endogenous latent mother–child interaction variable. Finally, a combined fifth SEM was run with attachment experiences scales Loving and Neglecting (Fig. 2).

All four models converged in ≤ 40 iterations and all indicators revealed significant parameter estimates (at *p* < 0.05) in the measurement models as well as good model fit (all SRMR < 0.04, RMSEA < 0.001, and CFI > 0.999). Two of these models revealed significant relationships between the latent variables: For Loving as the indicator of maternal (MAE) and paternal attachment experiences (PAE) the PAE reveal a significant path to EAS that is not present for MAE. Similarly, the SEM with Neglecting as the indicator, the path from PAE to EAS is significant while it is not for MAE. No such relationships are observed for Rejecting as indicator or Pressure to achieve (Supplement B). In a combined SEM with Loving and Neglecting attachment experiences with mother or father as indicators for MAE resp. PAE, the same relationships hold (Fig. 2). The path from PAE to EAS is significant, while the path from MAE to EAS is not, indicating that attachment experiences with the own father, but not with the own mother predict current mother–child behavior. A positive relationship between PAE and EAS indicates that more Loving experiences and fewer Neglecting experiences had with their own father are related to more positive mother–child interactions, i.e., more sensitive, less hostile, and less intrusive behavior toward the own child.

## Discussion

In general, the predicted relationships between own attachment experiences in childhood with both primary caregivers (mother and/or father) and current sensitive, non-intrusive and non-hostile mother–child interaction were revealed in this clinical sample. These results seem capable of contributing to our understanding of the transmission gap, as fewer loving experiences had with the father in childhood, i.e., the current grandparent generation, but also experiences of rejection, neglection and pressure to achieve are related to less sensitive or more intrusive and hostile behavior toward the own child in the present. While we found no evidence that RF moderates these effects, the exploratory structural equation modeling approach was able to deepen these findings by revealing that the paternal effect on emotional availability is larger than that of attachment experiences had with the own mother.

Nearly 60% of the participating mothers show an insecure or unresolved attachment classification (Table 1). This indicates the presence of cumulative risk factors in the sample, like having an own history of harsh life, trauma or missing positive parenting experiences. Such risk factors are known to lead to maternal insensitivity [28]. Maternal traumata have adverse effects on the ability to read the emotional signals of the child and to respond appropriately and sensitively to the child [29]. In turn, such insensitive behavior increases the child’s risk for adverse emotional and insecure attachment development [1].

### Mothers' own childhood experience and current emotional parenting

Our findings further show that mothers' experiences with their own both caregivers in the past are related to current emotional available behavior toward the child. For example, Loving childhood experiences with each caregiver are positively related to sensitive mother–child interactions. The remembered parental love is defined as having memories of a parent who is affectionate, accepting and supportive [25] and together with sensitive caregiving it might foster the own child’s secure attachment development in the next generation. As we can show with the present analyses, sensitive behavior toward the child might be a reasonable factor contributing to this transmission (although child attachment has not been the focus of the present examinations). We further found that unfavorable experiences like rejection and neglection by the own father are associated with less sensitive, and more hostile behavior toward the child. Also, perceived neglection by the own mother reduces sensitive mother–child behavior. Research revealed that a child’s attachment security is predicted by the pattern of ‘inner working models’ that caregivers have about their own attachment history [7]. The transmission of unfavorable behavior to the child can thus be explained by these models. In the present analyses, such transmission over 3 generations from the grandparents' generation (1st) to the mothers in the sample (2nd) and their behavior toward the child (3rd generation) was revealed. With less loving experiences and more experiences of neglection and rejection inner working models of negative memories might be present and seem to lead to current (less) emotional parenting.

### Intrusiveness and pressure to achieve

Of some interest might be the result that experienced pressure to achieve by the father is positively correlated with non-intrusive behavior. Intrusiveness is defined as being overprotective and/or interrupting the child’s activity undermining the child’s autonomy [24]. Pressure to achieve is characterized as pushing the child to succeed in general, or to take on adult responsibilities with punishment or withdrawal of affection if failed [25]. A mother who was pressured to achieve during her own childhood might transmit these patterns to the child and also pushes her child earlier into autonomy (e.g., is less controlling but more demanding). It can be discussed that she is therefore less intrusive during the interaction with the child. This result was not replicated in the moderation analyses. To rule out that the finding was spurious, the effect needs to be followed-up in future studies with a larger sample size.

### Role of reflective functioning

RF as a measure of mentalizing capacities is in the present study positively related to Loving attachment experiences and negatively with Rejection in their own childhood with both caregivers as well as negatively with experiences of Neglection by the father. A previous study examined this relation between rejecting experiences, RF and actual behavior in romantic partnerships [10]. The present findings extend these results in that they refer to the transgenerational aspect of behavior from the grandparent toward the parent and child (via the current mother–child interaction) by examining the relationship between RF and past attachment experiences. A higher RF was found to be associated with positive parenting like sensitive and less intrusive mother–child interactions. In the literature, RF is seen as a predictor for the quality of parent–child relationships and associated with positive and sensitive parenting [30]. Mothers with a high capacity to mentalize show less intrusive behavior [16]. Our findings extend these observations in that they point to maladaptive parenting because of difficult childhood experiences that also explains variance in RF (Table 3).

Unexpectedly, we did not find evidence for the buffering effects of RF on these relationships—neither for positive nor for maladaptive attachment experiences had with their own mother or father. The capacity to mentalize may increase the awareness of the child’s internal mental states (as indicated by correlations between RF and sensitivity and non-intrusiveness) but may not necessarily indicate the mothers’ ability to convert her thoughts about the infant’s mind into sensitive interactions with the child [1]. Especially in situations of heightened arousal the ability to mentalize and to provide consistent and sensitive caregiving might collapse. It has therefore been recommended to evaluate maternal behavior within the context of distress in order to tap into the behavioral characteristics that are most closely related to intergenerational transmission [16]. The videotaped free play interactions in the present study might not expose the dyad to this kind of distress which might explain the present results (c.f. limitations). RF might be more present at a representational level as it is shown in the videotaped interactions. With the relatively small sample size the power to find a moderator effect was low. We were not able to document the role of RF in these relationships between past attachment experiences and current mother–child behavior.

### Latent predictors of the mothers' emotional availability

The SEM approach—although exploratory—completes the above observations by underlining the stronger role that experiences had with the own father have in explaining current maternal emotional availability visible in mother–child interactions. More positive Loving and less maladaptive Neglecting attachment experiences with the father enhance a latent variable of emotional availability. And this latent variable is indicated by higher sensitivity, and lower intrusiveness and lower hostility in interaction with the child. Although maternal and paternal attachment experiences correlate to a substantial extent (*β* = 0.72), no such relationship for experiences had with the own mother is able to explain emotional availability. A distinct role of attachment experiences with their father explains present warm and non-hostile behavior toward the child. A result which mirrors recent findings that self-reported connectedness of fathers with their children explains lower aggressive tendencies in their children, while such a relation has not been observed for mothers [31]. It also points to studies showing that secure attachment to fathers and mothers is related to differential levels of cortisol reactivity of the child [32]. Hence, a differential role of mothers and fathers in affect and aggression regulation with buffering roles of secure attachment to either the father or the mother or both might exist and are in need of further examinations. While the role of such positive and warm experiences in childhood is known and has contributed to the definition of the transmission gap for children’s attachment development, it was mainly examined in relation to the own mother [7] or has not been differentiated between both parents [15].

### Strengths and limitations

This study was conducted based on a clinical sample of mother–child dyads suffering from mental health problems. To our knowledge, no studies have investigated the potential effect of the past attachment experiences on current interactions with the child using direct assessments. A further strength is the differentiation between past attachment experiences with both caregivers.

Still, there are some limitations concerning methods and present analyses. First, results from our clinical sample cannot be generalized to all mother–child dyads. Also, general reflective functioning (RF) instead of the parental reflective functioning (PRF) has been used in our analyses. Both are operationalizations of mentalization [33]. It seems likely that a more general RF measure predicts sensitive behavior less accurately than PRF in context of mother–child interaction as the lack of effects in our moderation analyses showed. It has been discussed [34] that trauma- and relation-specific RF may inhibit the ability to mentalize in specific areas of trauma. Berthelot and Ensink [35] showed that mothers who experienced trauma in childhood and with low trauma-specific RF were significantly more likely to have infants with a disorganized attachment status, whereas mothers with high trauma-specific RF were more likely to have securely attached infants. Relation-specific RF regarding each caregiver might therefore be quite different, particularly if some experiences with one caregiver are more negative. Thus, relation-specific RF and trauma-RF might be more predictive than general RF. The lack of a moderating effect of RF in the current study might thus be due to the focus on general RF. It seems reasonable to assume that a general RF is insufficient to cover all relationships and it should be differentiated into a relation-specific RF based on the relationship with each caregiver. To contribute to the transmission gap, trauma- and relation-specific RF may better explain the risk of intergenerational transmission of attachment. For future research, it also seems important to focus on RF in relation to each caregiver or attachment experience-specific RF (e.g., trauma-RF). A further limitation regards the AAI interview itself. The AAI experience scales are not assumed to reflect veridical reports of earlier experiences with caregivers, but rather to capture adults' depiction of earlier experiences at the time of the interview, thus, they may reflect mood-related biases. In addition to that, we could not address the attachment status of the children with the present data set. Furthermore, we could not provide the IRR for the coded EAS and AAI-scorings which limits our findings and ability to provide the agreement of the ratings.

We further limited our findings by not considering any parameters of the child. The reciprocity of interaction and the idea that the children may also show dysregulation in his/her emotional responses to the caregiver were neglected in this study. However, mentalization and the perception of the child are known to be influenced by the child’s responsiveness and character traits [5]. Furthermore, the analysis is based on a relatively small sample size and limited to mothers only. Research shows that fathers and mothers differ in how they form attachment relationships with their children and in co-parenting [36]. With the increasingly more active role of fathers and its importance in child development it seems recommended to consider the father’s role in children's attachment development and transmission of the father's past childhood experiences.

While we neglected the present role of fathers in parent–child interaction, the present results highlight the impact of past attachment experiences with fathers. Future research should therefore focus also on the triadic competences, as this is a relatively stable parameter [6]. The sample size might have impacted the SEM approach. It is recommended that SEMs should be based on larger samples (with a median of 200 cases) [27]. Because we relied on a highly select sample of mothers and children who were part of larger intervention studies one cannot simply increase sample sizes. On the other hand, sample size mainly effects the power of χ^2^-test statistics and the computation of standard errors. To limit these potential effects, fit indices that are less sensitive to sample sizes like RMSEA and SRMR and robust standard errors were reported [27]. We also limited the number of parameters of the SEMs to support model identification. The observed good model fit indices support these considerations. Even with this in mind, SEMs based on larger and probably also non-clinical samples are recommended to replicate and extend the present analyses.

## Conclusion

Our results suggest that mothers of a clinical sample with mental health problems seemed to show greater disruptive interactions with their young children if they experienced adverse attachment experiences with their parents or caregivers, especially with their father. We found that loving and neglecting attachment experiences with the father in childhood are predictors for the present interaction with the child and that these are transmitted to the ongoing relationship with the child. Hence, such results provide further insights into transgenerational transmission from the grandparents' generation to the present parent generation. The father’s role seems to be a key factor in this transmission, which is often not considered. These findings call for further research investigating the trajectories of attachment experiences including fathers and its future impact on the child and child development. A deeper understanding of the pathways for intergenerational transmission of risk and the processes that underpin transmission of attachment is needed and results showed again that the impact of the early interpersonal experiences is central to explain present behavior toward the child.

We found no evidence of a moderating role of RF in this transmission. The transmission of past attachment experiences and the idea of positive parenting (e.g., warmth, protectiveness, autonomy) and the family triad should be integrated in future research to understand how to prevent the transmission of mental health problems and a malignant attachment pattern.

### Supplementary Information

Below is the link to the electronic supplementary material.

Supplementary file1 (PDF 612 KB)

## Data Availability

The datasets used and analyzed during the current study are available from the corresponding author on reasonable request.

## Abbreviations

AAI: Adult Attachment Interview
RF: Reflective functioning
RCT: Randomized controlled trial
SKKIPPI: [Acronym] Parent-Infant-Psychotherapy in cohort- and intervention studies [Eltern-Säugling-Kleinkind-Psychotherapie mittels Prävalenz- und Interventionsstudien]
PIP: Parent–Infant Psychotherapy
EAS: Emotional Availability Scale
M.I.N.I.: Mini International Neuropsychiatric Interview
C1: Caregiver 1 = mother
C2: Caregiver 2 = father
SEM: Structural Equation Model
WLSMV: Weighted Least Squares Mean- and Variance-adjusted
MAE: Maternal attachment experiences
PAE: Paternal attachment experiences
CFI: Comparative Fit Index
RMSEA: Root Mean Square Error of Approximation
SRMR: Standardized Root Mean Square Residual
IRR: Inter-rater Reliability
