# Supplementary material for: Past attachment experiences, the potential link of mentalization and the transmission of behavior to the child by mothers with mental health problems: cross-sectional analysis of a clinical sample
Source: Eur Child Adolesc Psychiatry. 2023 Sep 5;33(6):1883–94. doi: 10.1007/s00787-023-02291-9 (PMC11211145; doi:10.1007/s00787-023-02291-9)
Supplement: Supplementary file 1 — Supplementary file1 (PDF 612 KB) [file 787_2023_2291_MOESM1_ESM.pdf]

Online Supplement

Title: Past attachment experiences, the potential link of mentalization and the transmission of behavior to the child by mothers with mental health problems: cross-sectional analysis of a clinical sample

Journal name: European Child & Adolescent Psychiatry

Authors: Mattheß, Janna [1,2], Koch, Gabriele[1], Keil, Thomas[3,4,5], Roll, Stephanie[3], Berghöfer, Anne[3], Ludwig-Körner, Christiane[1], Schlensog-Schuster, Franziska[2,6], Sprengeler, Mona Katharina[2], von Klitzing, Kai[2] & Kuchinke, Lars[1]

[1] International Psychoanalytic University, Berlin, Germany

[2] Department of Child and Adolescent Psychiatry, Psychotherapy and Psychosomatics, University of Leipzig, Leipzig, Germany

[3] Institute of Social Medicine, Epidemiology and Health Economics, Charité – Universitätsmedizin Berlin, Berlin, Germany

[4] Institute of Clinical Epidemiology and Biometry, University of Wuerzburg, Wuerzburg, Germany

[5] State Institute of Health I, Bavarian Health and Food Safety Authority, Erlangen, Germany

[6] University Hospital of Child and Adolescent Psychiatry and Psychotherapy, University of Bern, Bern, Switzerland

Corresponding author: Janna Matthess, International Psychoanalytic University, Stromstr. 3b, 10555 Berlin, Germany

E-Mail: janna.matthess@ipu-berlin.de

Online Supplement A: Results of the Moderatoranalyses
Online Supplement B: Results of the Structural Equation Modeling approach

Online Supplement A

Results of the 10 moderator analyses with RF (reflective functioning) as moderator and the attachment experiences scales (Loving, Rejection, Neglection, Pressure to achieve and Involving) as predictors and the subscales of the Emotional Availability Scales (Sensitivity, Non-hostility and Non-intrusiveness) as criterion. None of these analyses revealed a moderation effect, but of some interest is a trend for a moderation effect of RF on the effect a Neglecting father has on maternal Sensitivity (b= .045, p= 0.067; Table B4 & B4.1). Main effects of the predictors mainly mirror the correlation analysis.

Table A1. Moderatoranalysis with AAI attachment experiences scale Loving of mother and Sensitivity and RF as moderator

Moderation Estimates

			95% Confidence Interval			
	**Estimate**	**SE**	**Lower**	**Upper**	**Z**	**p**
Loving C1	0.1314	0.0577	0.00481	0.23327	2.28	0.023
RF	0.0724	0.0617	-0.04408	0.20149	1.18	0.240
Loving C1 ✻ RF	-0.0367	0.0241	-0.08615	0.00720	-1.52	0.129

Note. Loving C1 = loving of mother; RF= reflective functioning, * p < .05

Table A2. Moderatoranalysis with the AAI attachment experiences scale Loving of father and Sensitivity and RF as moderator

Moderation Estimates

			95% Confidence Interval			
	**Estimate**	**SE**	**Lower**	**Upper**	**Z**	**p**
Loving C2	0.1661	0.0608	0.0463	0.2802	2.733	0.006
RF	0.0627	0.0556	-0.0551	0.1726	1.128	0.259
Loving C2 ✻ RF	-0.0232	0.0261	-0.0710	0.0319	-0.889	0.374

Note. Loving C2 = loving of father; RF= reflective functioning, * p < .05

Table A3. Moderatoranalysis with the AAI attachment experiences scale Neglection of mother and Sensitivity and RF as moderator

Moderation Estimates

			95% Confidence Interval			
	**Estimate**	**SE**	**Lower**	**Upper**	**Z**	**p**
Neglecting C1	-0.1163	0.0616	-0.22283	0.0113	-1.890	0.059
RF	0.0965	0.0552	-0.00854	0.2115	1.750	0.080
Neglecting C1 ✻ RF	0.0298	0.0315	-0.02597	0.1019	0.945	0.344

Note. Neglecting C1 = Neglection of mother; RF= reflective functioning, * $p < .05$

Table A4. Moderatoranalysis with the AAI attachment experiences scale Neglection of father and Sensitivity and RF as moderator

Moderation Estimates

			95% Confidence Interval			
	**Estimate**	**SE**	**Lower**	**Upper**	**Z**	**p**
Neglecting C2	-0.1449	0.0494	-0.23762	-0.0458	-2.94	0.003
RF	0.0702	0.0577	-0.03859	0.1891	1.22	0.224
Neglecting C2 ✻ RF	0.0451	0.0240	-0.00344	0.0975	1.88	0.060

Note. Neglecting C2 = Neglection of father; RF= reflective functioning, * $p < .05$

Table A4.1 Simple Slope Analysis of the moderatoranalysis with Neglection of father and Sensitivity Simple Slope Estimates

			95% Confidence Interval			
	**Estimate**	**SE**	**Lower**	**Upper**	**Z**	**p**
Average	-0.1362	0.0501	-0.231	-0.0356	-2.718	0.007
Low (-1SD)	-0.2236	0.0685	-0.352	-0.0861	-3.264	0.001
High (+1SD)	-0.0489	0.0678	-0.177	0.0961	-0.721	0.471

Note. shows the effect of the predictor (Neglecting C2= father) on the dependent variable (EAS Sensitivity) at different levels of the moderator (RF)

Table A5. Moderatoranalysis with the AAI attachment experiences scale Loving of mother and Non-hostility and RF as moderator

Moderation Estimates

			95% Confidence Interval			
	**Estimate**	**SE**	**Lower**	**Upper**	**Z**	**p**
Loving C1	0.0443	0.0384	-0.0274	0.1199	1.1529	0.249
RF	0.0468	0.0370	-0.0203	0.1236	1.2643	0.206
Loving C1 ✻ RF	-6.82e−4	0.0156	-0.0327	0.0292	-0.0439	0.965

Note. Loving C1 = loving of mother; RF= reflective functioning, * p < .05

Table A6. Moderatoranalysis with the AAI attachment experiences scale Loving of father and Non-hostility and RF as moderator

Moderation Estimates

			95% Confidence Interval			
	**Estimate**	**SE**	**Lower**	**Upper**	**Z**	**p**
Loving C2	0.09490	0.0366	0.0208	0.1661	2.5914	0.010
RF	0.02309	0.0294	-0.0303	0.0819	0.7854	0.432
Loving C2 ✻ RF	-0.00109	0.0155	-0.0337	0.0292	-0.0704	0.944

Note. Loving C2 = loving of father; RF= reflective functioning, * p < .05

Table A7. Moderatoranalysis with the AAI attachment experiences scale Rejection of father and Non-hostility and RF as moderator

Moderation Estimates

			95% Confidence Interval			
	**Estimate**	**SE**	**Lower**	**Upper**	**Z**	**p**
Rejecting C2	-0.06810	0.0322	-0.1307	-0.00325	-1.922	0.055
RF	0.03380	0.0339	-0.0342	0.10090	0.997	0.319
Rejecting C2 ✻ RF	-0.00539	0.0146	-0.0345	0.02366	-0.369	0.712

Note. Rejecting C2 = rejection of father; RF= reflective functioning, * p < .05

Table A8. Moderatoranalysis with the AAI attachment experiences scale Neglection of father and Non-hostility and RF as moderator

Moderation Estimates

			95% Confidence Interval			
	**Estimate**	**SE**	**Lower**	**Upper**	**Z**	**p**
Neglecting C2	-0.08740	0.0415	-0.17314	-0.0111	-2.105	0.035
RF	0.05310	0.0353	-0.00152	0.1341	1.505	0.132
Neglecting C2 ✻ RF	-0.00331	0.0183	-0.03196	0.0419	-0.181	0.856

Note. Neglecting C2 = neglection of father; RF= reflective functioning, * p < .05

Table A9. Moderatoranalysis with the AAI attachment experiences scale Loving of mother and Non-intrusiveness and RF as moderator

Moderation Estimates

			95% Confidence Interval			
	**Estimate**	**SE**	**Lower**	**Upper**	**Z**	**p**
Loving C1	0.0758	0.0770	-0.0912	0.2112	0.985	0.325
RF	0.1228	0.0803	-0.0245	0.2854	1.528	0.126
Loving C1 ✻ RF	-0.0151	0.0330	-0.0818	0.0541	-0.459	0.646

Note. Loving C1 = loving of mother; RF= reflective functioning, * p < .05

Table A10. Moderatoranalysis with the AAI attachment experiences scale Pressure to achieve of father and Non-intrusiveness and RF as moderator

Moderation Estimates

			95% Confidence Interval			
	**Estimate**	**SE**	**Lower**	**Upper**	**Z**	**p**
Pressure to achieve C2	0.1195	0.0941	-0.0674	0.303	1.270	0.204
RF	0.0469	0.0691	-0.0923	0.180	0.678	0.498
Pressure to achieve C2 ✻ RF	0.0181	0.0432	-0.0655	0.106	0.419	0.675

Note. Pressure to achieve C2 = pressure to achieve of father; RF= reflective functioning, * p < .05

Online Supplement B

Structural Equation Modeling with AAI scales Loving, Rejecting, Neglecting and Pressure to achieve in relation to each caregiver as observed indicators of maternal and paternal attachment experiences and EA subscales Sensitivity, Non-hostility and Non-intrusiveness as indicators of the endogenous latent mother-child interaction variable.

Table B1. Structural Equation model to predict maternal EAS variable by Loving attachment experiences with both caregivers.

Parameters estimates

				95% Confidence Intervals				
**Dep**	**Pred**	**Estimate**	**SE**	**Lower**	**Upper**	**β**	**z**	**p**
EAS	MAE	0.109	0.125	-0.1356	0.354	0.103	0.874	0.382
EAS	PAE	0.281	0.109	0.0666	0.496	0.265	2.568	0.010

Note. Displayed are β estimates. Endogenous variables: EAS= Sensitivity, Non-hostility & Non-intrusiveness; Exogenous variables: PAE= AAI attachment experiences with father, Loving; MAE= AAI attachment experiences with mother, Loving. RMSEA < .001, CFI = 1.0, SRMR = .019

Table B1.1. Measurement model of the Structural Equation model to predict maternal EAS variable by Loving attachment experiences with both caregivers.

				95% Confidence Intervals				
**Latent**	**Observed**	**Estimate**	**SE**	**Lower**	**Upper**	**β**	**z**	**p**
MAE	Loving C1	2.175	0.0991	1.981	2.369	1.000	21.94	< .001
PAE	Loving C2	1.864	0.1015	1.665	2.063	1.000	18.37	< .001
EAS	Sensitivity	1.058	0.0841	0.893	1.223	0.991	12.59	< .001
	Non-hostility	0.436	0.0744	0.290	0.582	0.708	5.86	< .001
	Non-intrusiveness	0.932	0.0985	0.739	1.125	0.741	9.47	< .001

Note. Displayed are β estimates. Endogenous variables: EAS= Sensitivity, Non-hostility & Non-intrusiveness; Exogenous variables: PAE= AAI attachment experiences with father, Loving C2; MAE= AAI attachment experiences with mother, Loving C1.

Table B2. Structural Equation model to predict maternal EAS variable by Rejecting attachment experiences with both caregivers.

Parameters estimates

				95% Confidence Intervals				
**Dep**	**Pred**	**Estimate**	**SE**	**Lower**	**Upper**	**β**	**z**	**p**
EAS	MAE	-0.0173	0.116	-0.244	0.209	-0.0168	-0.150	0.881
EAS	PAE	-0.2431	0.124	-0.486	2.93e-4	-0.2358	-1.964	0.050

Note. Displayed are β estimates. Endogenous variables: EAS= Sensitivity (variance were set .001), Non-hostility & Non-intrusiveness; Exogenous variables: PAE= AAI attachment experiences with father, Rejecting; MAE= AAI attachment experiences with mother, Rejecting. RMSEA < .001, CFI = 1.0, SRMR = .035

Table B2.1. Measurement model of the Structural Equation model to predict maternal EAS variable by Rejecting attachment experiences with both caregivers.

				95% Confidence Intervals				
**Latent**	**Observed**	**Estimate**	**SE**	**Lower**	**Upper**	**β**	**z**	**p**
MAE	Rejecting C1	2.442	0.0983	2.249	2.635	1.000	24.85	< .001
PAE	Rejecting C2	2.462	0.1066	2.253	2.671	1.000	23.10	< .001
EAS	Sensitivity	1.118	0.0731	0.975	1.262	1.000	15.31	< .001
	Non-hostility	0.447	0.0782	0.294	0.600	0.699	5.71	< .001
	Non-intrusiveness	0.933	0.0942	0.749	1.118	0.721	9.91	< .001

Note. Displayed are β estimates. Endogenous variables: EAS= Sensitivity, Non-hostility & Non-intrusiveness; Exogenous variables: PAE= AAI attachment experiences with father, Rejecting C2; MAE= AAI attachment experiences with mother, Rejecting C1. Because the initial SEM estimation led to negative variance estimations for EA sensitivity this variance was fixed at .001. This had no effect on the overall model results in particular the PAE-EAS path was not significant in both models.

Table B3. Structural Equation model to predict maternal EAS variable by Neglecting attachment experiences with both caregivers.

Parameters estimates

				95% Confidence Intervals				
**Dep**	**Pred**	**Estimate**	**SE**	**Lower**	**Upper**	**β**	**z**	**p**
EAS	MAE	-0.0816	0.111	-0.299	0.1353	-0.0768	-0.737	0.461
EAS	PAE	-0.3123	0.117	-0.541	-0.0838	-0.2942	-2.678	0.007

Note. Displayed are β estimates. Endogenous variables: EAS= Sensitivity, Non-hostility & Non-intrusiveness; Exogenous variables: PAE= AAI attachment experiences with father, Neglecting; MAE= AAI attachment experiences with mother, Neglecting. * p < .05, RMSEA < .001, CFI = 1.0, SRMR = .010

Table B3.1. Measurement model of the Structural Equation model to predict maternal EAS variable by Neglecting attachment experiences with both caregivers.

				95% Confidence Intervals				
**Latent**	**Observed**	**Estimate**	**SE**	**Lower**	**Upper**	**β**	**z**	**p**
MAE	Neglecting C1	1.934	0.1755	1.590	2.278	1.000	11.02	< .001
PAE	Neglecting C2	2.138	0.1575	1.829	2.447	1.000	13.58	< .001
EAS	Sensitivity	1.031	0.0919	0.851	1.211	0.963	11.22	< .001
	Non-hostility	0.439	0.0861	0.271	0.608	0.704	5.11	< .001
	Non-intrusiveness	0.973	0.1160	0.746	1.200	0.772	8.39	< .001

Note. Displayed are β estimates. Endogenous variables: EAS= Sensitivity, Non-hostility & Non-intrusiveness; Exogenous variables: PAE= AAI attachment experiences with father, Neglecting C2; MAE= AAI attachment experiences with mother, Neglecting C1.

Table B4. Structural Equation model to predict maternal EAS variable by Pressure to achieve attachment experiences with both caregivers.

Parameters estimates

				95% Confidence Intervals				
**Dep**	**Pred**	**Estimate**	**SE**	**Lower**	**Upper**	**β**	**z**	**p**
EAS	MAE	-0.0215	0.106	-0.229	0.186	-0.0213	-0.203	0.839
EAS	PAE	0.1360	0.144	-0.147	0.419	0.1349	0.941	0.347

Note. Displayed are β estimates. Endogenous variables: EAS= Sensitivity, Non-hostility & Non-intrusiveness; Exogenous variables: PAE= AAI attachment experiences with father, Pressure to achieve; MAE= AAI attachment experiences with mother, Pressure to achieve. RMSEA < .001, CFI = 1.0, SRMR = .014

Table B4.1. Measurement model of the Structural Equation model to predict maternal EAS variable by Pressure to achieve attachment experiences with both caregivers.

Latent	Observed	Estimate	SE	95% Confidence Intervals Lower	Upper	β	z	p
MAE	Press to achieve C1	1.937	0.1436	1.655	2.218	1.000	13.49	< .001
PAE	Press to achieve C2	1.527	0.1739	1.187	1.868	1.000	8.78	< .001
EAS	Sensitivity	1.072	0.1185	0.840	1.305	0.988	9.05	< .001
	Non-hostility Non-	0.423	0.0825	0.261	0.696	0.696	5.12	< .001
	intrusiveness	0.909	0.1089	0.695	1.122	0.710	8.34	< .001

Note. Displayed are β estimates. Endogenous variables: EAS= Sensitivity, Non-hostility & Non-intrusiveness; Exogenous variables: PAE= AAI attachment experiences with father, Pressure to achieve C2; MAE= AAI attachment experiences with mother, Pressure to achieve C1.

Table B5. Structural Equation model to predict maternal EAS variable by Loving & Neglecting attachment experiences with both caregivers.

Parameters estimates

Dep	Pred	Estimate	SE	95% Confidence Intervals Lower	Upper	β	z	p
EAS	MAE	0.0128	0.191	-0.3609	0.387	0.0117	0.0673	0.946
EAS	PAE	0.4338	0.179	0.0838	0.784	0.3966	2.4295	0.015

Note. Displayed are β estimates. Endogenous variables: EAS= Sensitivity, Non-hostility & Non-intrusiveness; Exogenous variables: PAE= AAI attachment experiences with father, Loving & Neglecting; MAE= AAI attachment experiences with mother, Loving & Neglecting. RMSEA < .001, CFI = 1.0, SRMR = .030

Table B5.1. Measurement model of the Structural Equation model to predict maternal EAS variable by Loving & Neglecting attachment experiences with both caregivers.

Latent	Observed	Estimate	SE	95% Confidence Intervals Lower	Upper	β	z	p
MAE	Loving C1	1.925	0.2188	1.496	2.354	0.857	8.810	< .001
	Neglecting C1	-1.290	0.2384	-1.757	-0.823	-0.665	-5.41	< .001
PAE	Loving C2	1.754	0.1615	1.437	2.070	0.919	10.86	< .001
	Neglecting C2	-1.580	0.2191	-2.010	-1.151	-0.735	-7.21	< .001
EAS	Sensitivity	1.004	0.0991	0.810	1.198	0.969	10.13	< .001
	Non-hostility	0.434	0.0892	0.260	0.609	0.716	4.87	< .001
	Non-intrusiveness	0.931	0.1150	0.706	1.157	0.759	8.10	< .001

Note. Displayed are β estimates. Endogenous variables: EAS= Sensitivity, Non-hostility & Non-intrusiveness; Exogenous variables: PAE= AAI attachment experiences with father, Loving & Neglecting C2; MAE= AAI attachment experiences with mother, Loving & Neglecting C1.
